# Supplementary material for: A longitudinal evaluation of localised chronic Pseudomonas aeruginosa infection in cystic fibrosis rat models
Source: BMC Microbiol. 2026 Mar 10;26:368. doi: 10.1186/s12866-026-04893-0 (PMC13085380; doi:10.1186/s12866-026-04893-0)
Supplement: Supplementary file 3 — Supplementary Material 3. [file 12866_2026_4893_MOESM3_ESM.docx]

# Supplementary Data


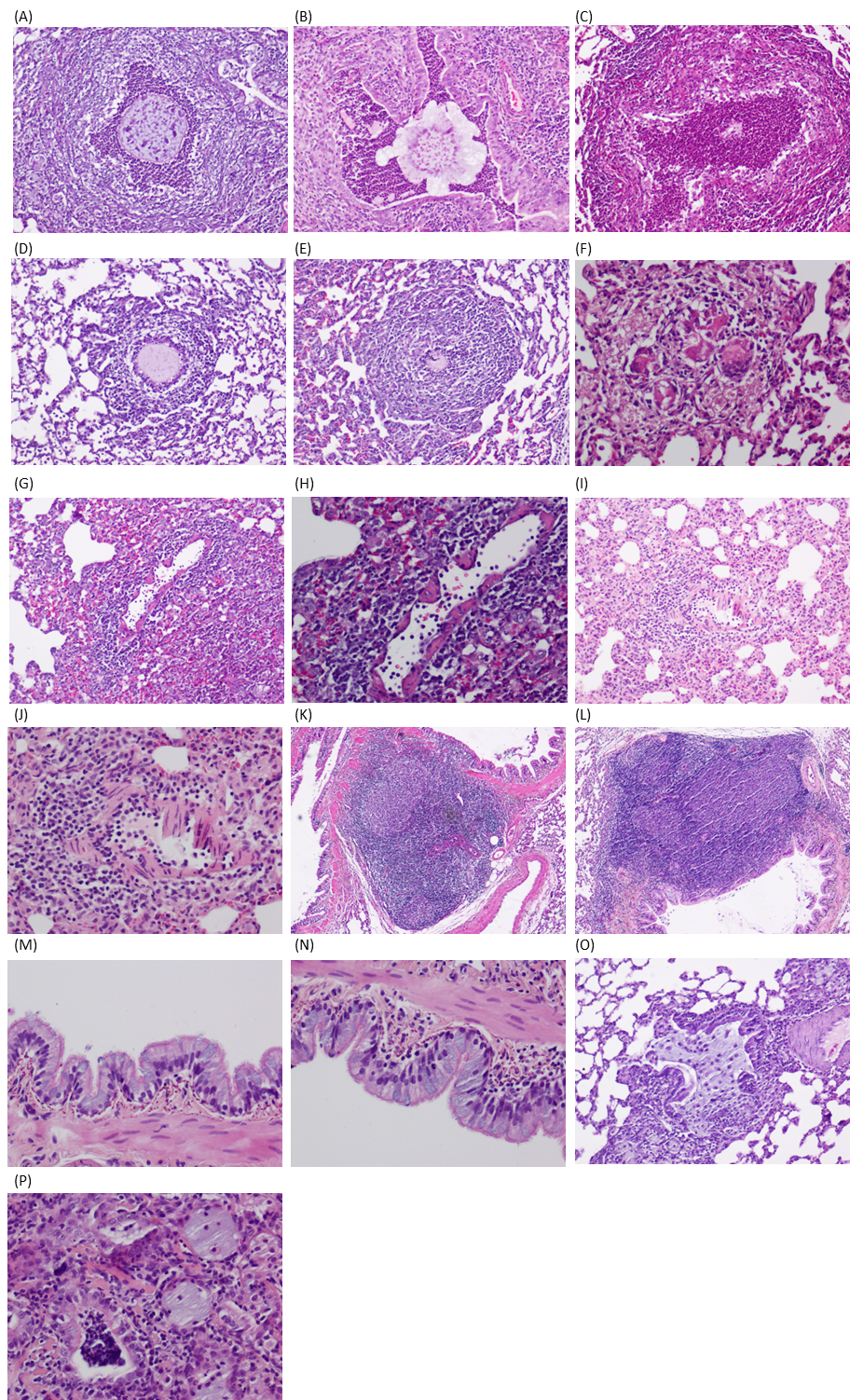


**Supplementary Figure 1: Detailed images of haematoxylin and eosin histology post-infection. (A)** *Phe508del* 7 days: A bacteria (arrows)-laden bead in a bronchiole is surrounded by abundant neutrophils. The necrotizing inflammatory reaction has almost completely destroyed the airway wall. **(B)** wildtype 7 days: A bronchus contains a partially disintegrated agar bead, with the lumen filled by abundant neutrophils (acute suppurative bronchopneumonia). **(C)** *Phe508del* 14 days: A bronchial lumen contains numerous neutrophils, with almost complete dissolution of the agar bead. There is severe destruction of the airway wall. **(D, E)** *Phe508del* 7 days: the agar bead is surrounded by macrophages and the severely damaged airway wall is heavily infiltrated by lymphocytes. **(F)** *Phe508del* day 31: Small, disintegrated fragments of the agar bead are being phagocytosed by multinucleated giant cells. **(G)** *Phe508del* 7 days: Severe lymphocytic vasculitis with perivascular lymphocytic cuffing. **(H)** *Phe508del* 7 days: Higher power view of showing many luminal lymphocytes with lymphocytic infiltration, and segmental destruction, of the blood vessel wall, which shows fibrinoid necrosis. **(I)** knockout day 21: Lymphocytic vasculitis and perivascular lymphoplasmacytic cuffing. Surrounding alveolar walls are thickened by infiltrating chronic inflammatory cells. **(J)** Knockout day 21: Higher power view of (I). **(K,L)** Knockout day 21: Bronchial-associated lymphoid tissue (BALT) hyperplasia, with prominent germinal centres in lymphoid follicles. **(M,N)** Knockout day 21 and *Phe508del* 7 days: The bronchial lining shows goblet cell hyperplasia, with mucus extrusion into the lumen evident from some of these cells. **(O)** *Phe508del* 7 days: A bronchiolar lumen contains abundant mucus, with attendant macrophages. **(P)** *Phe508del* day 21: Several bronchiolar lumina are filled with mucus, while a bronchiole (left of image) contains luminal necrotic cellular debris, admixed with neutrophils.

**Supplementary Table 1: Distribution of animal numbers by genotype, sex, and infection time point.**

| **Genotype** | **Timepoint** | **Male** | **Female** | **Total** |
| --- | --- | --- | --- | --- |
| Wildtype | D07 | 5 | 4 | 9 |
|  | D14 | 6 | 6 | 12 |
|  | D21 | 5 | 5 | 10 |
|  | D63 | 3 | 4 | 7 |
| *Phe508del* | D07 | 5 | 4 | 9 |
|  | D14 | 3 | 5 | 8 |
|  | D21 | 6 | 4 | 10 |
|  | D63 | 4 | 3 | 7 |
| Knockout | D07 | 1 | 6 | 7 |
|  | D14 | 3 | 4 | 7 |
|  | D21 | 9 | 2 | 11 |
|  | D63 | 3 | 5 | 8 |
